# SBEAMS-Microarray: database software supporting genomic expression analyses for systems biology

**DOI:** 10.1186/1471-2105-7-286

**Published:** 2006-06-06

**Authors:** Bruz Marzolf, Eric W Deutsch, Patrick Moss, David Campbell, Michael H Johnson, Timothy Galitski

**Affiliations:** 1 Institute for Systems Biology, 1441 N. 34^th ^Street, Seattle, Washington, USA

## Abstract

**Background:**

The biological information in genomic expression data can be understood, and computationally extracted, in the context of systems of interacting molecules. The automation of this information extraction requires high throughput management and analysis of genomic expression data, and integration of these data with other data types.

**Results:**

*SBEAMS-Microarray*, a module of the open-source Systems Biology Experiment Analysis Management System (*SBEAMS*), enables MIAME-compliant storage, management, analysis, and integration of high-throughput genomic expression data. It is interoperable with the *Cytoscape *network integration, visualization, analysis, and modeling software platform.

**Conclusion:**

*SBEAMS-Microarray *provides end-to-end support for genomic expression analyses for network-based systems biology research.

## Background

The extraction of biological information from high throughput genomic expression data is a fundamentally network-based systems biology problem [1]. Complex cell properties such as pathogenicity, growth control, and metabolic capabilities arise from networks of molecular interactions. Control of such cell properties involves gene activity at multiple levels, including mRNA and protein levels, and molecular modifications, localization, and interactions. The computational integration of disparate network-data types and the application of network-analysis algorithms enables the extraction of information that is not contained in individual network elements or single data types.

Systems biology research is characterized by the development and application of technologies enabling quantitative measurements that are genomic in scale[2]. Data collected at multiple levels of gene activity are integrated, analyzed, and modeled to suggest further experiments in an iterative cycle of discovery. Microarray-based genomic expression measurement is the technology that comes closest to meeting the demands of systems biology research. Microarray-based measurement technologies are mature relative to proteomics and other genome-scale molecular interrogation methods. A major current bottleneck in systems biology is the extraction of biological information from genomic expression data integrated with other data types.

Before the development of *SBEAMS-Microarray*, existing microarray data-analysis software packages were evaluated based on desired attributes for the support of microarray-based systems biology research. These attributes included:

1. Free open-source availability

2. Compliance with the Minimum Information About a Microarray Experiment (MIAME) data content specification [3].

3. Flexible application of multiple microarray-data analysis methods

4. Strong support for large-scale datasets derived from the high-probe-density Affymetrix microarray platform

5. Integration of genomic expression data with other data types such as proteomics data

6. Interoperability with network visualization, analysis, and modeling software, for example, the *Cytoscape *platform [4].

However no single package had all of these attributes. Several packages [5-8] were developed for two-color spotted arrays, and lack support for high-probe-density Affymetrix data. Other database software projects such as BASE 2 [9] intend to support Affymetrix data, but have not yet been released at the time of this publication. Other packages [10,11] require proprietary software and hardware, in these cases an Oracle database and Sun server. Finally, none of the evaluated packages [5-8,10-12] support integration of genomic expression data with other data types. Therefore, we developed the *Microarray *module of *SBEAMS*, the Systems Biology Experiment Analysis Management System [13]. *SBEAMS-Microarray *combines all of the above attributes, making *SBEAMS-Microarray *uniquely advantageous for network-based systems biology research.

## Implementation

### Architecture

**Figure 1:**
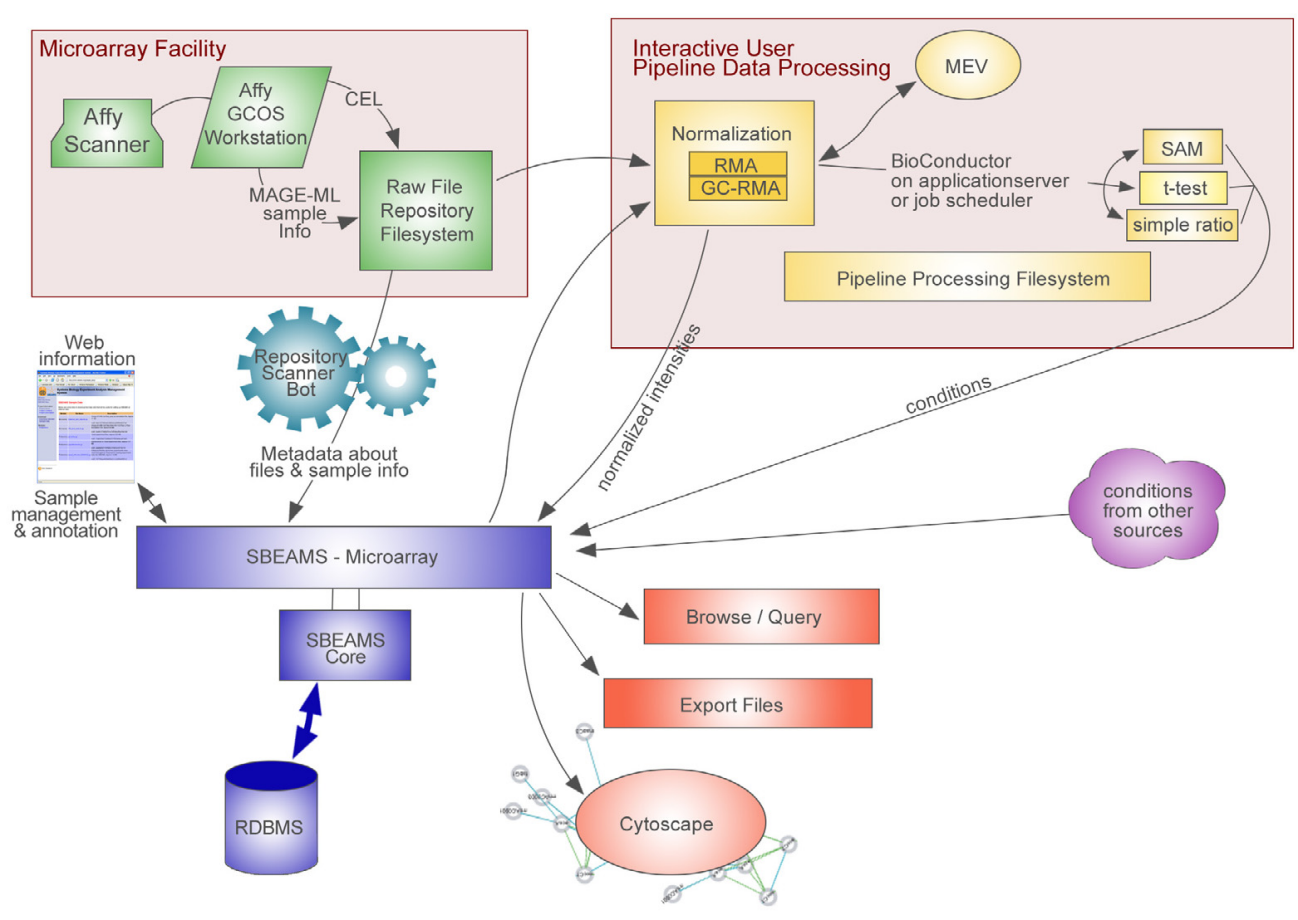
**Architecture of *SBEAMS-Microarray***. *SBEAMS-Microarray *is built using the *SBEAMS *Core framework, which provides tools to build a user interface with authentication and handles interactions with the back-end RDBMS. Affymetrix chip data are produced using vendor-provided equipment and software and placed in a raw file repository, where they are automatically imported by the *SBEAMS-Microarray *repository scanning component. Logged-in users may then annotate their data, run it through an interactive pipeline processing system, query the results, and export the data to various formats and visualization/analysis software packages.

The *SBEAMS-Microarray *module is built using *SBEAMS*, a software and database framework for collecting, storing, accessing and analyzing data from different types of experimental data [13]. *SBEAMS *combines a relational database management system (RDBMS) back end, a collection of tools to store, manage, and query experiment information and results in the RDBMS, a web front end for querying the database and providing integrated access to remote data sources, and an interface to other data processing and analysis programs (Figure 1).

### Client-server model

*SBEAMS*, including the *SBEAMS-Microarray *module, uses a web-based client-server software model. Thus, the *SBEAMS *software runs and is updated only on a central server. Also, computationally intensive tasks are handled on the server end. On the client end, the user needs only minimal computing power, a modern web browser with Java Web Start [14] installed, and a network connection to the *SBEAMS *server. Users connect to *SBEAMS *via HTTPS to the web server on which *SBEAMS *is installed. Perl CGI scripts use the *SBEAMS *API to create a web interface to the back-end database. The Perl DBI module is used for database connectivity. The system is designed to support any type of RDBMS for which a DBI driver is available. *SBEAMS-Microarray *is known to work with Microsoft SQL Server, Sybase Adaptive Server Enterprise, and MySQL, the most popular open-source RDBMS. Work is underway to add support for PostgreSQL, another popular open-source RDBMS.

### Database

The database schema definitions are provided in a database-independent format with the *SBEAMS *distribution. A script that generates data definition language (DDL) commands (i.e. CREATE TABLE, etc.) for the schema for several different RDBMSs is also provided. Its use is described in the installation instructions. A schema diagram (Additional file 1) depicts the *SBEAMS-Microarray *database module schema, including tables containing information about samples, arrays and associated files, intensity measurements, probe set annotations, analysis runs, and the final expression results. Additional tables containing information about *SBEAMS *users, work groups, permissions, projects, species, etc. are part of the *SBEAMS *Core schema and are not reproduced here.

### Installation

*SBEAMS-Microarray *and installation instructions can be downloaded as part of the latest release of *SBEAMS *at the *SBEAMS-Microarray *web site [15]. Alternatively, the Subversion [16] version control software can be used to obtain the current development version of *SBEAMS *from the Subversion repository. Subversion installation allows the software to be updated easily as new additions and changes are created, as well as allowing users who continue to extend *SBEAMS *through software development to contribute their work. Evaluation of *SBEAMS *prior to installation is available through a demonstration instance of the software at the *SBEAMS-Microarray *web site [15]. This site additionally provides access to two mailing lists, one for general questions and discussion, and the other for developers. *SBEAMS *and *SBEAMS-Microarray *are available under the terms of the GNU General Public License version 2.

### Security

Access to the interfaces and data in *SBEAMS-Microarray *is controlled through a comprehensive security model. Each user must log in to gain access to the database, and can belong to any number of work groups. A work group defines a set of privileges for each user in the work group. The software provides default administrative and user roles. Non-administrative users can create projects, and have full permissions for projects they own. By default, a user cannot view projects belonging to other users. However a user can grant varying levels of access to other users or work groups to facilitate sharing of data and analysis results. Specific result sets are shared easily by emailing hyperlinks to users that have access to a given project.

### Administration

After *SBEAMS-Microarray *is installed, administration is handled primarily through the web interface, with a set of tools available only to users with administrative privileges. These tools, accessible via menu choices visible only to such users, provide functionality such as creating new types of arrays, modifying records for users other than themselves, and deleting incorrect records. Other administrative functions, such as loading new array annotations, are handled at the command-line. These array annotations serve as the primary source of probe information during analyses conducted through *SBEAMS-Microarray*, and are loaded from quarterly updates that Affymetrix produces. Command-line functions are authenticated to enforce user permissions.

## Results and discussion

### Data types

*SBEAMS-Microarray *primarily supports the high-probe-density Affymetrix platform. All current Affymetrix gene-expression microarrays are supported, and all future Affymetrix arrays should be supported as well, given the generic mechanism for loading information about new array types. Two-color microarray support exists, however it is disabled by default, given that two-color microarray quantitation and annotation formats are not standardized. Modifications could be made to the software to support additional two-color microarray data formats, allowing the two-color portion of the software to be used.

### Data management

*SBEAMS-Microarray *functions seamlessly with the high-throughput high-probe-density Affymetrix microarray platform and GCOS system software [17] as shown in Figure 1. Microarray facility staff or end users enter information about their samples into the Affymetrix GCOS software. After scanning, data extraction, and initial processing, GCOS exports these data, with all of the raw data files, to an output data directory as a standard MAGE-ML file. *SBEAMS-Microarray *periodically scans the output data directory for new data sets, and automatically loads any complete sets into the *SBEAMS *database. The database stores information about each microarray and pointers to the locations of data files, which are stored in an *SBEAMS*-managed file tree. After automated data loading, users can access an overview of all their arrays in a web-based user interface, with the ability to view or download raw data files and data quality reports. Users can edit or add sample information and annotations to comply with the MIAME data content specifications [3].

### Data queries

*SBEAMS-Microarray *supports simple queries allowing quick access to data for genes of interest. Users specify a search string and select the arrays from which they want to see expression data and detection calls. The results are presented in a matrix of data with colored visual cues. MAS 5.0 signal values and detection calls [18] are used because normalization can be done on each microarray independently, whereas other normalization methods such as RMA [19] depend on the normalization group.

An advanced SQL query tool also exists, allowing more search parameters and resulting in tabular data output.

### Data analysis

*SBEAMS-Microarray *incorporates widely used open-source genomics softwares, and thereby supports the flexible application of multiple microarray-data processing and analysis methods. To perform processing tasks including background correction, normalization, probe set summarization and differential expression testing, we integrated the *BioConductor *[20] open-source web interface package, *webbioc*, into *SBEAMS-Microarray*. The *webbioc *package implements several processing methods, including RMA [19], GC-RMA [21], VSN [22], MAS 5.0 [18], dChip [23], Quantile normalization [24] and Qspline normalization [25]. Processing may occur on the *SBEAMS *server or may be submitted to a batch scheduler on a computer cluster. Email notification of completed processing jobs is available. A data-processing summary page provides several diagnostic plots to help in the identification of microarrays that failed or are inconsistent with the rest of the data set, and links to download the processed data. After processing of a specific data set, *SBEAMS-Microarray *supports differential-expression testing with three optional methods: simple ratio analysis, t-test, and the SAM false-discovery-rate method [26]. SAM and t-test are available for data sets with replicate experiments, and produce test statistics for each probe set as well as a view of the probe sets with the highest scores and accompanying annotations. After a data analysis has been performed, the user can elect to load the results into the database where it can be stored and queried, or integrated with other data types.

*SBEAMS-Microarray *incorporates the *MultiExperiment Viewer *(*MeV*), developed at The Institute for Genomic Research. *MeV *provides numerous statistical tests, classification methods, and clustering algorithms [8] that extend the analytical capabilities of *SBEAMS-Microarray*. From the *SBEAMS-Microarray *web interface, *MeV *is launched using Java Web Start, so that users do not need to install *MeV*, and are immediately presented with their data in the *MeV *environment.

### Data integration

*SBEAMS *is modular in design to allow the integrated storage and access of disparate types of experiments and data, for example, microarray and proteomics experiments, molecular interaction data, and gene annotations. This integrated system is a consistent framework that combines a RDBMS back end, and a web front end providing integrated access to the data. For example, from the *SBEAMS-Microarray GetExpression *interface, queries can be made for gene annotations of interest or by defining threshold levels of metrics for statistical significance of expression change across one or more user-specified microarray experiments. The results can be viewed within the *SBEAMS *web interface, or exported in Excel, CSV, TSV, or XML formats, or accessed programmatically via HTTPS. Data sets may originate in-house or be imported from external sources. Currently, development of *SBEAMS *is driven mainly by the *SBEAMS-Microarray *and *SBEAMS-Proteomics *projects, with multiple modules in early stages of development.

*SBEAMS-Microarray *is interoperable with *Cytoscape *software. The results of data analyses can be loaded directly to the *Cytoscape *environment, launched from the *SBEAMS *environment. *Cytoscape *is an open-source bioinformatics software platform for visualizing molecular interaction networks and integrating these networks with gene expression data, proteomics data, gene annotations, and other data [4]. A wide variety of additional functionalities are available as *Cytoscape *plugins. Plugins implement integrated network analyses, connection with outside databases and tools, and modeling capabilities. In *SBEAMS-Microarray*, query results from *GetExpression *can be loaded directly into *Cytoscape *via Java Web Start, as described above for *MeV*. With the data in *Cytoscape*, users can load molecular interaction data, annotation data, and a wide variety of other data, to generate integrated networks. Data types can be loaded directly as files, or imported from outside databases using plugins like *InteractionFetcher *[27].

Interoperability with *Cytoscape *enables automated data integration and subsequent network-based analyses to extract information that is not present in any one data type. For example, the *Biomodules *plugin [28] implements methods for the computational identification of groups of interacting proteins performing some collective function (modules) in integrated networks of genomic expression data, molecular interaction data, and gene annotation data. Prinz et al. [28] applied these methods to discover and experimentally validate molecular insights on the regulation of yeast cell differentiation from the familiar yeast form to the filamentous-invasive form.

### Intended use

*SBEAMS-Microarray *enables investigators to store, manage, analyze, and integrate genomic expression data for systems biology research projects. Investigators begin by performing a microarray experiment to answer questions about their biological system of interest. Once the primary data have been obtained, investigators log in through the *SBEAMS-Microarray *web interface to see their data that has been automatically loaded into the database. Before beginning analysis, it is advisable to ensure that the data are acceptable by viewing various quality control metrics and diagnostic plots provided by the software. Investigators may choose to annotate their data further, by providing greater details on the biological samples hybridized to their microarrays to aid others involved with analysis of their data and in compliance with MIAME standards [3]. Once satisfied with their data quality, investigators may begin to gather biological information by using the querying interfaces to inspect the expression patterns of one or more genes with known (or potentially interesting) responses under their experimental conditions. After establishing confidence in their expression data, investigators use the data analysis pipeline. After applying one of several optional normalization methods, investigators have the option to launch seamlessly the *MultiExperiment Viewer *[8], which provides multiple methods to cluster and visualize the data. A second option, producing results that ultimately will lead to data integration and network visualization and analysis in *Cytoscape*, is differential expression analysis. Investigators choose parameters for their analysis, including a statistical method, groups of biological replicates to be compared, and thresholds for statistically significant differential expression. After analysis, result tables show genes with the greatest and most significant expression differences. Users have the option to store the results in the database. These tables of differentially expressed genes are themselves informative, but will provide further system-level insights when integrated with other biological data. From stored analyses, sets of genes with their differential expression values can be loaded directly into *Cytoscape *launched from *SBEAMS*. Expression data can be mapped to interpolated colors of nodes representing differentially expressed genes. Investigators then may begin to use *Cytoscape *to explore their expression data in the context of biological network information. One method is to employ the *InteractionFetcher *[27] plugin to find and integrate data on interactions among the differentially expressed genes and their products. As noted above (Data integration), the integration of these interaction data, and other data types, with the expression data produces networks that investigators analyze to find relationships that are not contained in either data type alone. Cytoscape enables integration of many data types (e.g., proteomics data, annotation data, etc.), customizable visualization of these integrated data, and computational analysis of integrated networks to extract system-level information on the questions motivating the study (e.g., refs [27] and [28]). Uses of the software are detailed in the *SBEAMS-Microarray User Guide *(Additional file 2).

*SBEAMS *is an open-source software project. Its design is intended to facilitate further development. *SBEAMS *allows multiple separate instances of the software to be installed on the same machine, so that software developers may have one or more developmental versions where they improve or extend *SBEAMS-Microarray*, without interfering with the production instance of the software. Developmental improvements are tested and eventually added to the code repository and rolled out to the production instance.

### Future development

Implementation enhancements are planned. Active development is underway to support other backend database software such as PostgreSQL, so that SBEAMS – Microarray can be implemented on the local RDBMS of choice.

Support for MAGE-OM/ML database standards [29] is planned. Use of these standards will allow interoperability of *SBEAMS-Microarray *with other MAGE-compliant software and allow creation of MAGE-ML documents for submission of experimental data to public repositories such as *ArrayExpress *[30].

A major goal for *SBEAMS-Microarray *development is the addition of support for more types of microarrays and experimental assays. Development will be required to support the emergence of new arrays and platforms, particularly with respect to integrating results across different generations, and possibly platforms, of microarrays. Support for experiments based on genome-tiling microarrays is a priority. These microarrays enable high-throughput genome-scale investigations of alternative splicing, non-coding RNA levels, protein-DNA interaction, and comparative genomics. These assays require new data analysis methods, e.g., [31], which will be incorporated.

Additional tools for integrating analysis results from separate *SBEAMS *modules are planned. Currently *SBEAMS *allows for storage, access and analysis of disparate data types in their respective modules within the *SBEAMS *framework. External tools such as *Cytoscape *must be used to integrate these multiple data types. An interface for combining microarray, proteomics and interaction data within *SBEAMS *is currently under development.

## Conclusion

*SBEAMS-Microarray *is a useful tool for both a microarray facility and its diverse user community. It is uniquely strong in its flexible incorporation of multiple data analysis methods and supporting softwares, its support of data standards, its open-source availability, and its support for data integration and network analyses. *SBEAMS-Microarray *is a key module in the *SBEAMS *database system, which has several other modules (e.g., *SBEAMS-Proteomics*) allowing for incorporation of disparate data types into a single framework. In combination with network-analysis tools like *Cytoscape*, it provides end-to-end support for systems biology research projects involving high-throughput genomic expression analysis.

## Availability and requirements

• **Project name: **SBEAMS-Microarray

• **Project home page: **

• **Operating system(s):**

○ Application server: Linux/UNIX

○ RDBMS: Windows or Linux/UNIX

• **Programming languages: **Perl, SQL, PHP, JavaScript

• **Other requirements:**

○ Apache web server

○ R 2.1.0 through 2.3.0 with Bioconductor 1.6 through 1.8

○ Microsoft SQL Server, Sybase Adaptive Server Enterprise or MySQL

○ FreeTDS 0.63

○ libgd 2.0.33

• **License: **GNU General Public License version 2

• **Restrictions to use by non-academics: **None

## Authors' contributions

BM contributed to software design, did software testing and debugging, and drafted the manuscript. EWD conceived the software project, designed and coded software, and drafted the manuscript. PM designed and coded software. DC coded and debugged software. MHJ coded and debugged software. TG supervised the project, participated in software design, and revised the manuscript. All authors read and approved the final manuscript.

## Supplementary Material

Additional file 1 **Schema of *SBEAMS-Microarray***. A GIF image representation of the *SBEAMS-Microarray *database schema. The information used to generate this diagram, including detailed column definitions and relationships, is in the *SBEAMS *distribution which can be obtained at . Click here for file

Additional file 2 ***SBEAMS-Microarray *User Guide**. SBEAMS-Microarray_UserGuide.pdf provides instructions on using the various functions available in *SBEAMS-Microarray*. Click here for file
