# Supplementary figures and images for: SBEAMS-Microarray: database software supporting genomic expression analyses for systems biology
Source: BMC Bioinformatics. 2006 Jun 6;7:286. doi: 10.1186/1471-2105-7-286 (PMC1524999; doi:10.1186/1471-2105-7-286)

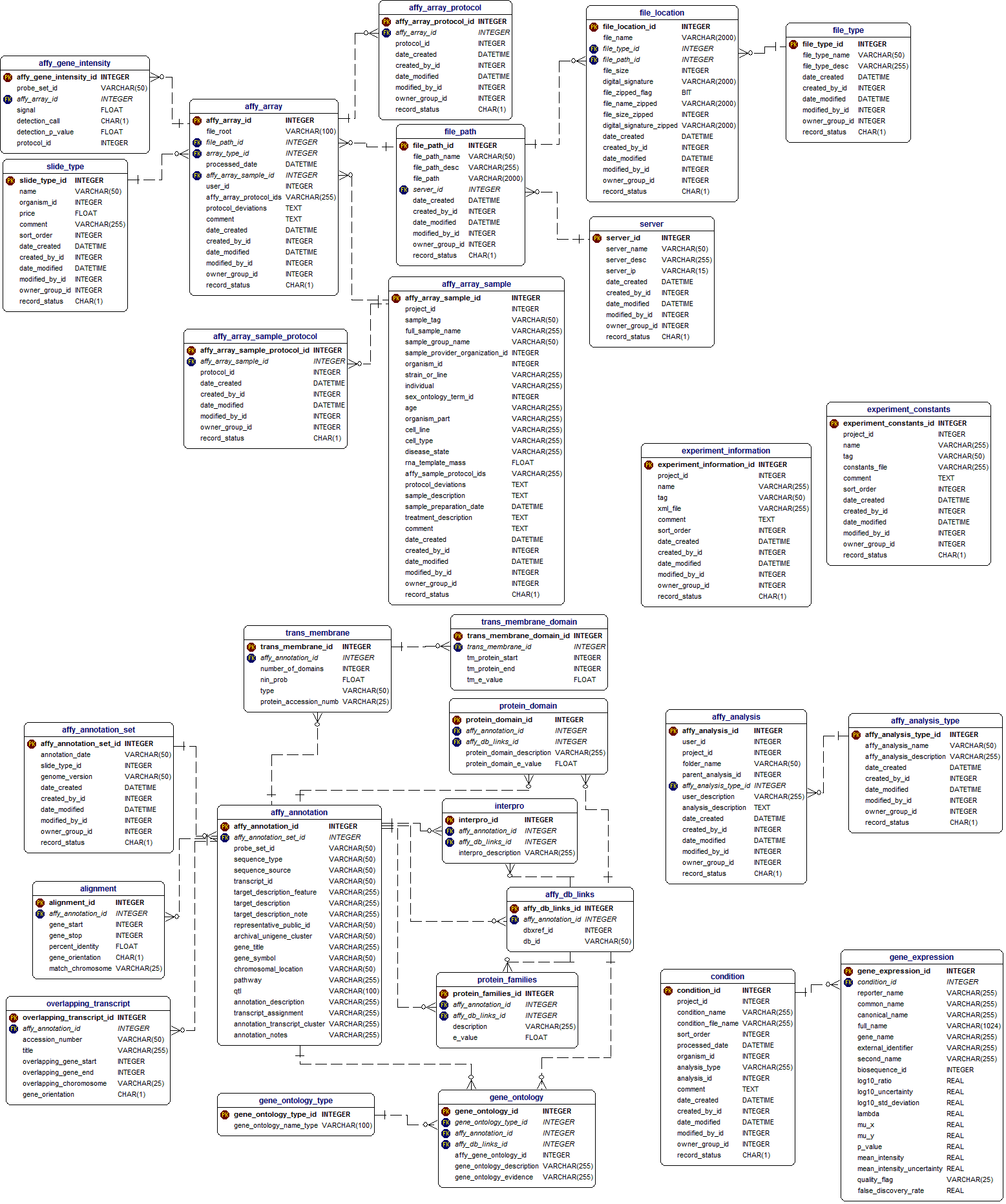

Supplement: Additional file 1 — Schema of SBEAMS-Microarray. A GIF image representation of the SBEAMS-Microarray database schema. The information used to generate this diagram, including detailed column definitions and relationships, is in the SBEAMS distribution which can be obtained at . [file 1471-2105-7-286-S1.png]
