# Supplementary material for: SBEAMS-Microarray: database software supporting genomic expression analyses for systems biology
Source: BMC Bioinformatics. 2006 Jun 6;7:286. doi: 10.1186/1471-2105-7-286 (PMC1524999; doi:10.1186/1471-2105-7-286)
Supplement: Additional file 2 — SBEAMS-Microarray User Guide. SBEAMS-Microarray_UserGuide.pdf provides instructions on using the various functions available in SBEAMS-Microarray. [file 1471-2105-7-286-S2.pdf]

SBEAMS-Microarray
User Guide

Bruz Marzolf
December 23, 2005

Table Of Contents

1 Basic Data Access 3

1.1 Getting Into *SBEAMS* 3
1.2 Download Array Data 3
1.2.1 Access the Download Page 3
1.2.2 File Types 4
1.3 Annotate Affy Samples or Arrays 5
1.3.1 Annotate Sample Page 5
1.3.2 Annotate Array Page 6
1.4 Simple Query 6
1.5 Advanced Query 7
1.5.1 Entering Query 7
1.5.2 Viewing Query Results 7

2 Analysis Pipeline 7

2.1 Normalization 7
2.1.1 Starting a New Analysis Session 7
2.1.2 Selecting and Grouping Arrays 8
2.1.3 Choosing Normalization Options 10
2.1.4 Viewing Results 10
2.2 Differential Expression Testing 11
2.2.1 Return to Normalization Set 11
2.2.2 Perform SAM Run 11
2.2.3 Adding data to GetExpression 12
2.3 Querying GetExpression and Launching Cytoscape 13
2.3.1 Query Data 13
2.3.2 Launch Cytoscape 13
2.3.3 Viewing Expression Data in Cytoscape 14

1 Basic Data Access

1.1 Getting Into *SBEAMS*

1) Login:

   a. http://<SBEAMS server name>/sbeams

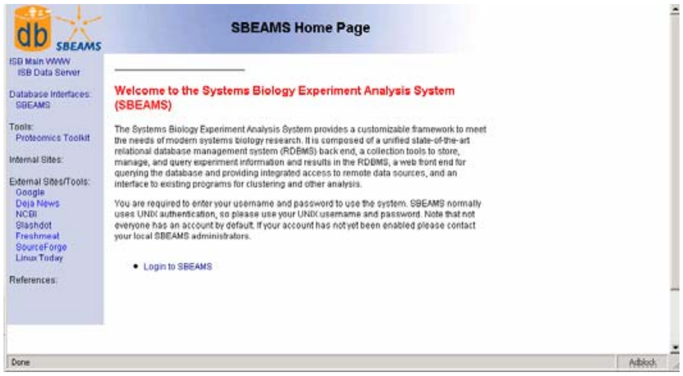

Figure 1.1 Login page for SBEAMS

   b. Click on the link **Login to SBEAMS**

   c. Login with your username and password as set up by your *SBEAMS* Administrator

2) Go to Microarray Module

   d. On the left menu bar click on **Microarray** to enter *SBEAMS-Microarray*

1.2 Download Array Data

Many array data files can be downloaded or viewed directly from *SBEAMS*

1.2.1 Access the Download Page

To download or view array files from a particular project choose the **Download Data** link in the left menu.

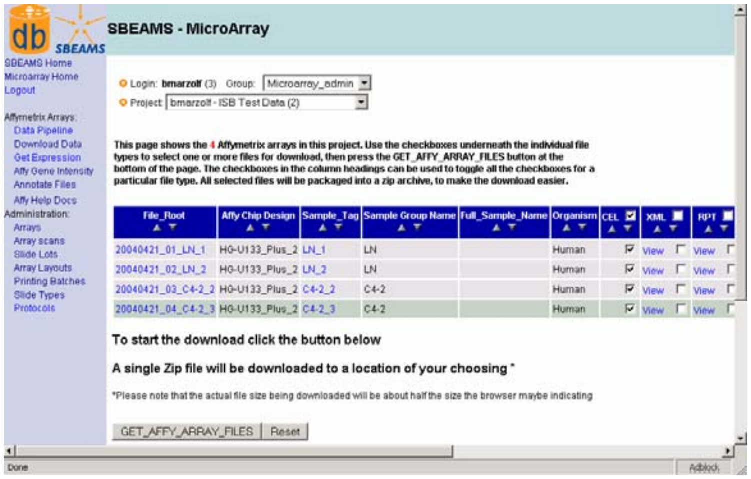

Figure 1.2 Download Data page

1) Select a project from the drop down list located at the top of the page.
2) To download files, select the check boxes for the file type you'd like for each array you'd like to download. To download one type of file for all arrays in the project, check the box next to the file type at the top of the table (e.g. CEL, RPT). The default setting is to download all the CEL files from a project.
3) Once all the files are selected click the button **Get_affy_array_files**
4) Select a location to save the zip file containing all the information selected for downloading

1.2.2 File Types

Here is a brief description of all the file types available for viewing or downloading from SBEAMS

File Extension	Description	View File Directly	Download
CHP	CHP. Binary Affymetrix file. CHP files contain probe set analysis results generated from Affymetrix software.	NO	Yes
CEL	CEL. Binary Affymetrix file. The CEL file stores the results of the intensity calculations on the pixel values of the DAT file	NO	Yes
XML	XML. MAGE XML Affymetrix file. Contains information from Affymetrix GCOS Software collected during sample preparation, hybridization, washing and scanning.	Yes	Yes

RPT	RPT. Text report. Contains information about the CHP file, used for basic quality control	Yes	Yes
R_CHP	R_CHP. Text File. Contains Probe set intensity values, calculated by using R/Bioconductor affy mas5.0 algorithms	No	Yes
JPEG	JPEG. Jpeg image of the Affy Chip generated by R using the image method within the affy library	Yes	Yes
EGRAM_PF.jpg	EGRAM_PF.jpg. Electrophoregram image of the Pre-fragmented cRNA	Yes	Yes
EGRAM_T.jpg	EGRAM_T.jpg. Electrophoregram image of the total RNA	Yes	Yes
EGRAM_F.jpg	EGRAM_F.jpg. Electrophoregram image of the fragmented cRNA	Yes	Yes

1.3 Annotate Affy Samples or Arrays

All samples hybridized to Affymetrix arrays can be annotated and viewed within SBEAMS.

1.3.1 Annotate Sample Page

To access the page to annotate or view information about a sample, choose the **Microarray Home** link in the left menu.

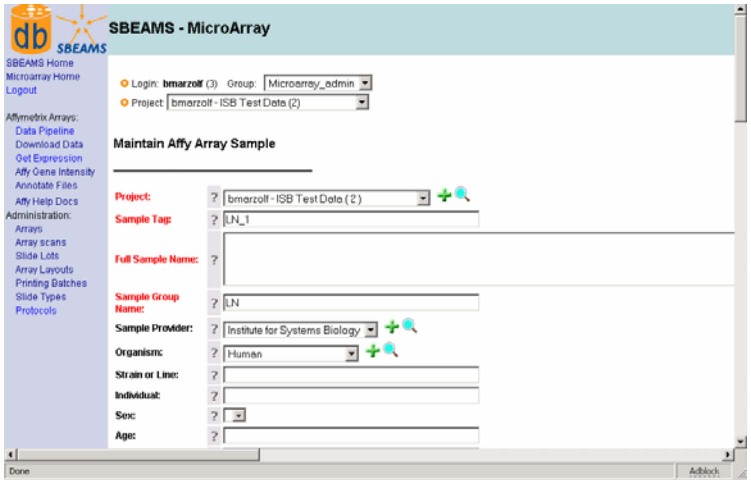

Figure 1.3 Annotating a Sample

1) Select a project from the drop down located at the top of the page.
2) Click on a Link under the “Sample_Tag" Column to view information about a sample

3)	Enter Data to describe the sample

4)	To save time entering multiple samples use the "Save Template" functionality. This will save a copy of the current page. To re-use the template on your next sample, choose the template in the "Existing Templates" drop down box. Then click **Set Fields to this Template**. Change any fields that are different for the second sample.

5)	Click "Insert" if this is a new record or "Update" if the record was modified to save the information

1.3.2 Annotate Array Page

To access the page to annotate or view information about a specific Affy Array, choose the **Microarray Home** link on the left menu.

1)	Select the project from the drop down located at the top of the page.

2)	Click on a Link under the "File_root" Column to view information about an array

3)	Enter Data to to describe the array

4)	Click "Insert" or "Update" to save the information

1.4 Simple Query

Perform simple queries within a project to view probe set intensity values. Expression values are taken from the R_CHP files.

Choose the **Affy Gene Intensity** link on the left menu to access the Simple Query page.

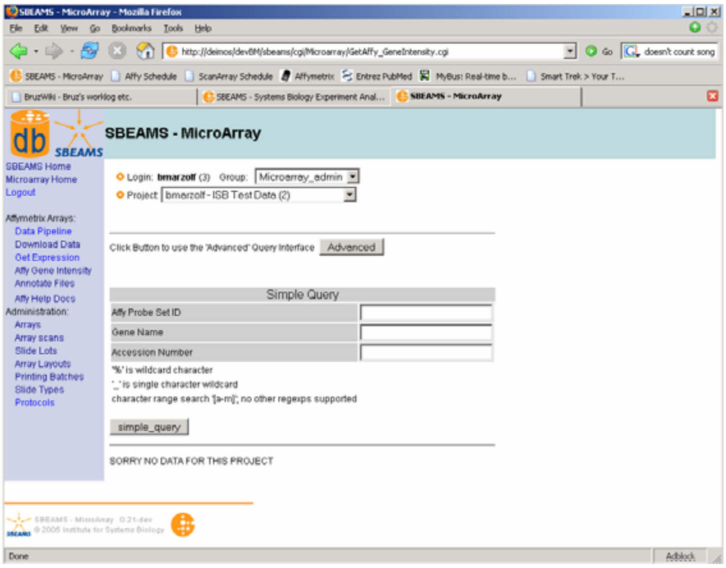

Figure 1.4 Simple Query interface

Enter a query with an Affy probe_set_id, Gene Symbol, Gene Name or Accession number:
1) Choose the project of interest
2) Select specific arrays, default is to have all arrays for the project selected
3) Enter the search term
4) Hit the "Simply_Query" button

1.5 Advanced Query

Perform Advanced queries within one or many projects to view probe set intensity values. Expression values are taken from the R_CHP files.

1.5.1 Entering Query

1) Change to the Advanced Query page, select one or more projects, arrays and query constraints:
2) Choose the project(s) of interest
3) Select the arrays in the chosen projects you wish to query
4) Enter search constraints such as the probe set ID, gene symbol
5) Select the Data Columns you want to display, such as Signal Intensity and/or Detection P-value
6) Select Display Options
7) Hit the “QUERY” button to run your query

1.5.2 Viewing Query Results

- Data can be displayed in a html table, tsv,csv,excel or xml formats
- Any of the columns my be sorted
- Link to Affy annotation page is provided

2 Analysis Pipeline

2.1 Normalization

Using the pipeline begins with starting a new analysis session, choosing and grouping arrays, and then picking normalization options.

2.1.1 Starting a New Analysis Session

1) Switch to a project of interest using the drop-down list at the top of the page.
2) Choose the **Data Pipeline** link on the left menu bar
3) Start a new Analysis Session
   a. Click “Start New Analysis Session”

2.1.2 Selecting and Grouping Arrays

The grouping page is broken into three sections: Arrays that have been selected on the **top** part of the page, projects to choose arrays from in the **middle** and arrays within a project on the **bottom**.

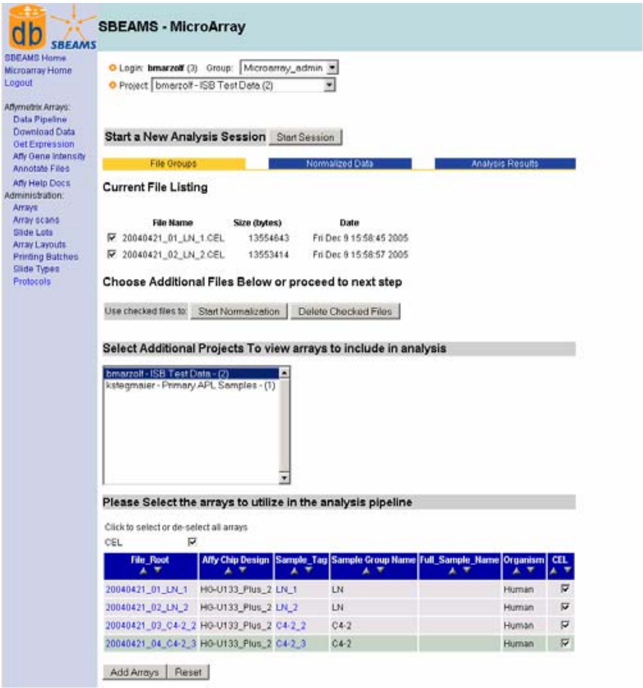

Figure 2.1 Adding files to a File Grouping

1) Multiple projects can be selected at the same time by clicking projects in the window “Select Additional Projects to view arrays to include in analysis”
2) Uncheck any arrays that should not be included in the analysis run.
3) Click the Add Arrays button at the bottom of the screen. The selected arrays will now appear at the top of the screen.
4) To add additional arrays, repeat steps 1-3.
5) Click the “Start Normalization” button

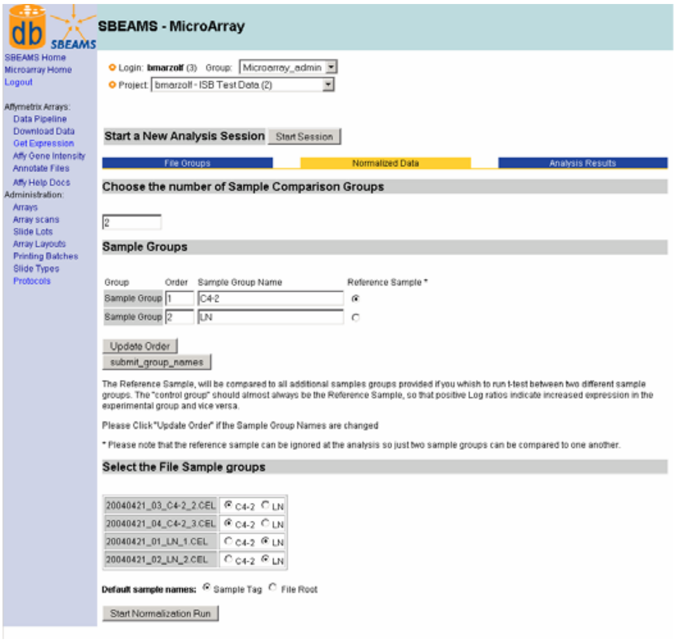

Figure 2.2 Choosing a reference sample, sample group names and assigning groups to each array in the File Grouping step

6) Add Sample Group information and select a reference sample. Replicate arrays will be combined together at the analysis phase if they are in the same sample group. The sample Group information is <u>NOT</u> utilized for any of the normalization calculations; it is simply collected here for use in further analyses that occur after normalization.
7) Change the number and names of groups as desired
8) Click "Update Order"
9) Associate CEL files with the appropriate Sample Group Names
10) Click "submit_group_names"
11) Make sure all the files are in the correct sample groups
12) Click "Start Normalization Run"

2.1.3 Choosing Normalization Options

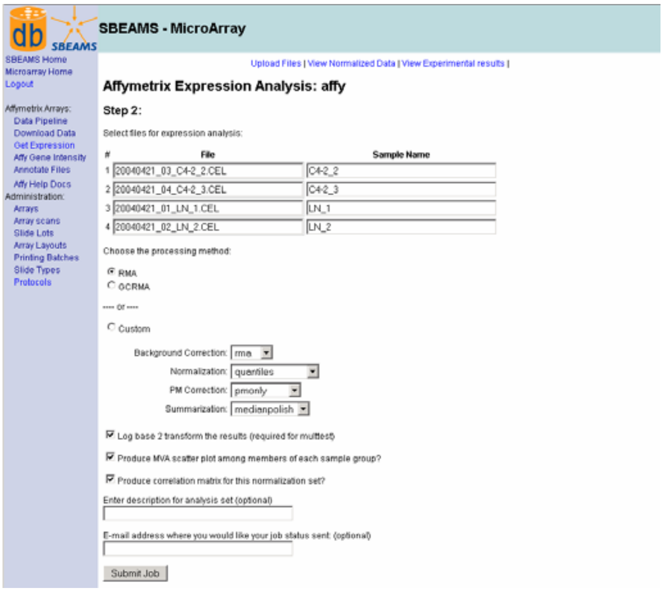

Figure 2.3 Normalization step options

1) You may edit the Sample names, which will be used to annotate the results
2) Select either RMA, GC-RMA or Custom
3) When Custom is chosen, you may separately choose the Background Correction, Normalization, PM Correction and Summarization options. These should be chosen with care as not all combinations are possible or desirable.
4) Optionally select whether Log base 2 results are desired (default, recommended)
5) Optionally select whether MVA plots and correlation matrices should be produced. These are useful as diagnostic plots. These require substantially more memory during analysis, and should be turned off to facilitate normalization of large numbers of arrays.
6) Optionally name the analysis (recommended to differentiate multiple analyses of the same data set)
7) Optionally enter an email address that will be sent a message upon completion of normalization run.
8) Click "Submit Job"

2.1.4 Viewing Results

1) Either return to the browser window where you performed the analysis, or click on the result link in the email sent to you by the pipeline.
    a. Click on the link **Show Files** located at the top of the page

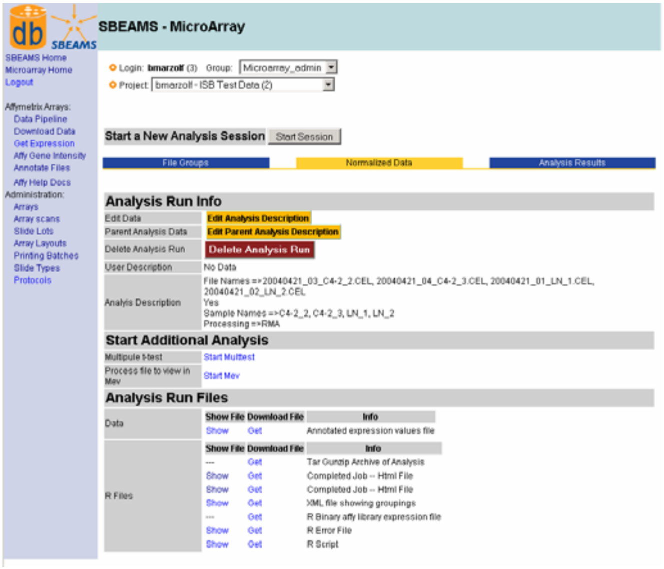

Figure 2.4 Viewing available options and data in Normalization results

2) View the normalized data file
   a. Under the header Analysis Run Files→Data click the link **Show**
   b. This load log2 expression values for all the genes and arrays just analyzed, although it's not very useful in the browser window.
3) To download the normalized data, under the header Analysis Run Files→Data click the link **Get**. These results may then be loaded into Excel or other software.

2.2 Differential Expression Testing

2.2.1 Return to Normalization Set

1) Make sure the project where you performed normalization is selected
2) Click **Data Pipeline** link on the left navigation menu
3) Click the "Normalization" tab
4) Click the **Show Files** Link for your normalization run

2.2.2 Perform SAM Run

1) Find the heading Start Additional Analysis->Multiple t-test
   a. Click the link **Start Multtest**
2) Select Analysis method. Default is to run SAM
   a. Leave the radio button selection at "SAM"
3) Click the button "Next Step"
4) Review the reference sample and sample groups to be analyzed. Make sure the information is correct.

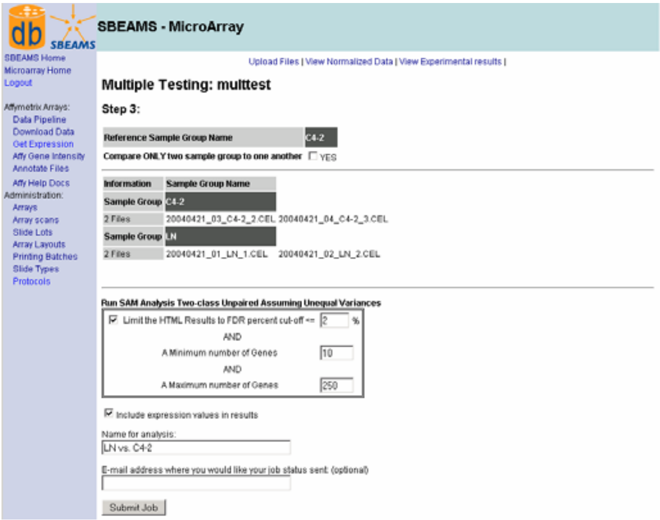

Figure 2.5 Options for SAM Analysis

   a. When the run starts the reference sample will be compared to the other experimental sample groups.
   b. A single pairwise comparison out of a set of many groups may be performed by selecting the checkbox labeled 'Compare ONLY two sample group to one another'
5) Review SAM options for limiting FDR cutoff, minimum and maximum genes
6) Optionally choose a "Name for analysis" for your results (recommended to differentiate multiple analysis runs).
7) Optionally add an e-mail address to be notified after the run is complete.
8) Click "Submit Job"

2.2.3 Adding data to GetExpression

In GetExpression, the data can be further analyzed, merged with different results sets and viewed in Cytoscape.

1) In your SAM or t-test result page, under Add Results to Get Expression→Add Data, choose **Add Data Link**
2) Click "Check Condition Names" to see whether this condition already exists. If the name is unique proceed to the next step, otherwise go back to the previous step and create a new name
3) Click the button "Upload Conditions." Wait for the data to load and go to the GetExpression page after the data is loaded
4) Click the link Go to Get Expression Page **here** at the bottom of the page OR choose the **GetExpression** link on the left menu bar

2.3 Querying GetExpression and Launching Cytoscape

2.3.1 Query Data

1) Select the desired project(s) – Conditions from multiple projects may be chosen.
2) Click the **GetExpression** link on the left menu bar
3) Within the Conditions menu box select the conditions of interest
4) The Following columns MUST be selected in order to ensure the data can be loaded into Cytoscape:
   Data Columns to Display:
   - Log 10 Ratio
   - False Discovery Rate

   Display Options:
   - Show All Conditions if one condition meets criteria
   - Pivot Conditions as columns
5) Click the “Query” button

2.3.2 Launch Cytoscape

6) Click the link next to Download ResultSet in Format:→ **Cytoscape**
7) When the web page come back, click the first three Links (The Boss must go first):
   - Boss
   - Network
   - Data Matrix Viewer

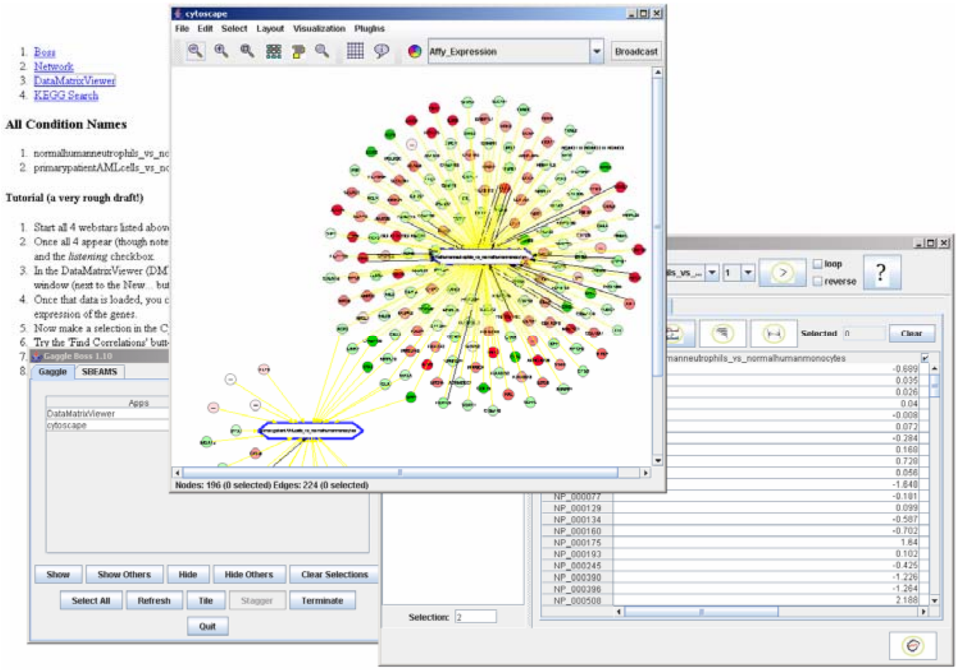

Figure 2.6 Java Web Start of Cytoscape, Boss, and Data Matrix Viewer

2.3.3 Viewing Expression Data in Cytoscape

1) Make sure all three programs are running: Gaggle Boss, Cytoscape and Data Matrix Viewer(DMV)
2) Load the Expression Data
    a. Click on the DMV window
    b. Click on the folder "Expression"
    c. Click on the button to the right of "new" 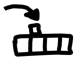
3) Load the expression data into Cytoscape
    a. Within the DMV go up to the drop down menu currently labeled "None", and select the first Condition in the list. This should make the circle (gene nodes) change color in Cytoscape
    b. To play a movie click the icon >
4) Manually Select the genes that both conditions share in common in Cytoscape
    a. Find the Cytoscape window
    b. Click and drag to highlight the gene nodes. Use Shift if all of the genes cannot be selected at one time
    c. Right click to view the gene names
5) Use a filter to select all the genes that both conditions have in common
    a. Click the "Grid" icon located at the top-middle of the Cytoscape menu bar
    b. Find the tab "Topology 1"
    c. Enter 2 in the field "Minimum Number of Neighbors"
    d. Enter 1 in the field "Within Depth"
    e. Click Select
6) Graph the Selected genes in the DMV to view the global expression profile
    a. Make sure no genes are selected in the DMV. Click the "Clear" button in the DMV window
    b. In Cytoscape with the genes of interest highlighted, click the "Broadcast" button, located in the upper right corner.
    c. Go to the DMV window.
    d. Select the Log10Ratio Tab. It should indicate that "7" Genes are selected.
    e. Click the icon 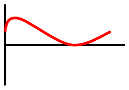
7) Find Genes that have a similar expression profile
    a. Make sure no genes are currently selected. Go to the Log10ratios window and click the "Clear" button
    b. Within the graph made in step 5, click a gene name with an interesting profile.

c. Click the “Broadcast” icon 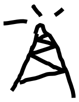 Located in the upper left hand part of the screen
d. Go back to the Log10ratio screen.
e. Click the icon 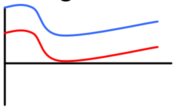
f. Move the slider so the “Threshold” reads 99
g. Graph the data by clicking the icon in 5-e
h. To highlight the genes in Cytoscape click the Broadcast button. 5-c
    i. Note that all the genes selected in the DMV my not be viewable in Cytoscape since the expression network is a small subset of all the expression data loaded from the GetExpression query.

8) Apply GO annotation
    a. Click the information icon top-middle of the Cytoscape menu
    b. Click on “Go, Molecular Function, Homo sapiens”
    c. Click on level “4”
    d. Click “Apply Annotation to All Nodes”
    e. Open the folder on the right half of the screen and walk through the different GO levels. Look at the Cytoscape screen to see what becomes highlighted.
